# Peer-led Stress Prevention Seminars in the First Year of Medical School – A Project Report

**DOI:** 10.3205/zma001002

**Published:** 2016-02-15

**Authors:** Till Johannes Bugaj, Christine Mücksch, Carolin Schmid, Florian Junne, Rebecca Erschens, Wolfgang Herzog, Christoph Nikendei

**Affiliations:** 1 University Hospital of Heidelberg, Department of General Internal and Psychosomatic Medicine, Heidelberg, Germany; 2 University Hospital of Tübingen, Department of Psychosomatic Medicine and Psychotherapy, Tübingen, Germany

**Keywords:** stress, psychosocial burden, medical studies, prevention

## Abstract

**Introduction: **From the beginning of the first year of medical studies, increased psychological stress and elevated burnout prevalence rates can be registered compared to sample populations. Characterized by learning “on an equal footing”, the principle of peer-assisted learning (PAL) is widely used in medical education. This report aims to showcase the development and evaluation of peer-led stress prevention seminars for first year medical students after one year of implementation.

**Project description: **With each of the three sessions lasting 90 min., the stress prevention seminars took place in small groups (6-10 students) in the period from November 2013 to January 2014 and from November 2014 to December 2014 at the Medical Faculty of Heidelberg. Led by trained peers, the seminar content ranged from psycho-educational elements, i.e. time management strategy development and test anxiety assistance, to relaxation techniques. All seminar sessions were evaluated via questionnaire. All questions were answered on a Likert scale ranging from 1 to 7 (1=strongly agree; 7=strongly disagree).

**Results: **75 students consented to participate in seminars (65% female; aged 20.5±3.3 years). The series of seminars was averagely given the school grade of 1.2±0.4 (1=very good to 6=unsatisfactory) in WS 2013/14 and 1.5±0.5 in the following year and the peer tutors’ competence was evaluated as very high (1.4 to 1.5 approval rate on the Likert scale).

**Discussion: **The seminar sessions’ importance to the students is underlined by their very positive evaluations. This offer seems to have benefited students especially during the demanding transitional phase at the start of their studies. Both the implementation of the preventive measure at an early stage as well as the use of PAL seem to have proven effective.

**Conclusion:** PAL seems to be effective in the field of stress prevention. However, specific efficacy studies are still lacking.

## Zusammenfassung

**Einleitung:** Ab dem ersten Studienjahr kommt es bei Medizinstudierenden zu einer kontinuierlichen Zunahme von psychischen Belastungen und folglich einer - gegenüber der vergleichbaren Bevölkerungsstichprobe - erhöhten Burnout-Prävalenz. Das Prinzip des peer-assisted learning (PAL) zeichnet sich durch „Lernen auf Augenhöhe“ aus und findet breite Anwendung im Studium der Humanmedizin. Ziel des vorliegenden Projektberichts ist die Vorstellung freiwilliger tutorengeleiteter Stresspräventions-Seminare für das erste Semester Humanmedizin sowie die Darstellung der Akzeptanzdaten nach einem Jahr der Durchführung.

**Projektbeschreibung:** Die dargestellten Seminare fanden im Zeitraum von November 2013 bis Januar 2014 sowie von November 2014 bis Dezember 2014 in Kleingruppen (6-10 Studierende) an jeweils drei Terminen à 90 min. an der Medizinischen Fakultät Heidelberg statt. Die Leitung der Seminare übernahmen geschulte studentische Tutoren/innen. Die Inhalte der Seminare reichten von psychoedukativen Elementen; über das Erarbeiten von konkreten Strategien zum persönlichen Zeitmanagement und Hilfestellungen bei Prüfungsangst; bis hin zur Anwendung von Entspannungsverfahren. Sämtliche Seminarabende wurden per Fragebogen evaluiert. Dabei wurden die Fragen jeweils auf einer Likert-Skala von 1 bis 7 (1=stimme voll zu; 7=stimme gar nicht zu) beantwortet.

**Ergebnisse: **Es nahmen 75 Studierende an den freiwilligen Seminaren teil. 65% der Teilnehmer/innen waren weiblich. Das Durchschnittsalter lag bei 20,5 (SD±3,3) Jahren. Die Seminarreihe wurde im Durchschnitt mit der Schulnote 1,2 (SD±0,4) im WS 2013/14 und 1,5 (SD±0,5) im Folgejahr bewertet. Dabei wurde die Kompetenz der studentischen Tutoren als sehr hoch eingestuft (Zustimmungen von 1,4 – 1,5 auf der Likert-Skala).

**Diskussion: **Die sehr guten Bewertungen der Seminarabende unterstreichen die Bedeutung für die Studierenden, die gerade in der Schwellenphase des Studienbeginns von diesem Angebot zu profitieren scheinen. Neben dem frühen Zeitpunkt der Präventionsmaßnahme scheint sich vor allem die Entscheidung für tutoriell geleitete Seminare bewährt zu haben.

**Schlussfolgerung:** PAL scheint sich auch für den Bereich der Stressprävention zu bewähren – allerdings existieren bisher keine spezifischen Wirksamkeitsstudien.

## Introduction

In the medical profession, the in part considerable psychosocial burden can not only result in lasting quality of life impairment in physicians but can also lead to an impairment of their patients’ medical care [1]. Compared to relevant population samples, increased burnout prevalence rates can be shown for physicians and already for medical students [2]. From the beginning of the first year of medical studies, increased psychological burden can be noted while health awareness simultaneously decreases [3], [4]. During the co-called “practical year” (PJ), which completes the degree course, a striking 20% of medical students have been shown to reach values in the Maslach Burnout Inventory (MBI) indicating elevated burnout risk [5]. Maslach et al. were able to show that these are accompanied by emotional exhaustion, increasing depersonalization and feelings of reduced personal accomplishment [6]. Maslach et al. define the experience of contact as emotionally overwhelming and the loss of the ability to regenerate as emotional exhaustion. Depersonalization means that physicians have negative, cynical attitudes and impersonal feelings toward their patients. Hence, Maslach et al. often refer to the diagnostic dimension as “cynicism”. The feeling of reduced personal accomplishment refers to the tendency to experience one’s own activity as insufficiently competent and/or effective [7]. Here, high performance demands as well as workload are primarily discussed as reason for stress. Although medical students have explicitly expressed their need for specific interventions for the prevention of psychosocial disorders [8], their multiple psychosocial burdens have only been discussed and increasingly addressed by medical faculties in recent years. Moreover, medical students’ mere perception of their faculty as actively trying to prevent student stress burden has been shown to reduce the students’ number of depressed days and suicidal ideation [9]. Furthermore, it has been shown that stress burden is reduced when universities actively raise awareness for burnout and emotional problems [10]. 

Several studies have examined the effects of relaxation techniques on medical students’ stress levels and were able to show them to impact on, for example, psychological symptoms (e.g. depression), perceived stress level, and cognitive parameters [11], [12], [13]. However, to our knowledge, studies on peer-led stress prevention seminars, differing significantly in content from mere mentoring concepts, for first year medical students are lacking so far.

The principle of peer-assisted learning (PAL), which was used in our peer-led stress seminars, enables students and tutors to “learn on an equal footing” [14], [15]. PAL has been proven, depending on the focus of the interventions, to support the cognitive, psychomotor and affective development of student learners which, in turn, have been shown to increase self-esteem, autonomy, clinical reasoning, self-evaluation and collaboration with like-minded peers [16]. On the other hand, student tutors benefit through the development of their personal knowledge, skills and attitude becoming, almost in passing, better learners themselves, and acquiring important skills for later activity as lecturers [17]. These effects have often been attributed to the tutors’ and students’ so-called “social and cognitive congruence” [18], [19]. The construct of social congruence implies that student tutors and students are able to communicate with each other informally and in a particularly empathetic manner because of their similar social roles [20]. In addition, cognitive congruence refers to the fact that student tutors and students share a similar level of knowledge and have comparable learning experiences. Accordingly, as both are able to speak the same “language” and meet at eye-level, explanations are given at an appropriate level [15]. 

The alarming data on stress burden in young medical students and the available data presented above on the effectiveness of stress management programs on the one hand, and the pedagogical concept of PAL on the other hand, served as starting points for the development of the peer-led stress prevention seminars for first semester medical students at the Medical Faculty of Heidelberg. This project report aims to

1. showcase the seminar concept and
2. evaluate first year medical student’s acceptance of the intervention program after one year of implementation.

As the concept of PAL was used, we hypothesized acceptance to be high and that PAL tutors were well suited to teach the specific content.

## Project Description

### Participants

All medical students who commenced their studies at the Medical Faculty of Heidelberg in the winter semester (WS) of 2013 and 2014 (WS 2013: n=321/WS 2014: n=338) were invited to participate in the peer-led stress prevention seminars on a voluntary basis. 

#### Setting and procedure 

The stress prevention seminars took place in small groups (6-10 students) on three evening appointments lasting 90 minutes each in the period from November 2013 to January 2014 and in the period from November to December 2014 in the following year at the Medical Faculty Heidelberg. Seminars were led by trained student tutors. All student tutors already had profound practical teaching experience, e.g. having taught as Aal^plus^- (Anatomie am Lebenden plus: Anamnese und Körperliche Untersuchung [Living Anatomy plus: history taking and physical examination]) or Skills-Lab-tutors previously [21], but were additionally intensively prepared by the first author of the present project report prior to both times of implementation. Student tutors’ preoperational training took place in the weeks before the start of the seminar in 2013 and 2014. Here, student tutors were able to experience the stress seminar series themselves as participants in “real time”. Further theoretical background and/or didactic issues were discussed after each session. Aside from the standardized predefined teaching content, student tutors were given the possibility to add relevant content at their discretion. Accordingly, student tutors were encouraged, for example, to share their own coping experiences with the sometimes demanding university life in the seminars. Each seminar session included teaching of basic knowledge on various aspects of stress, stress management and time management, as well as discussion and small group work among students to promote in-depth processing of the seminar content [22]. The aim was to create a trusting atmosphere which would enable students to talk about possible stressors, discuss suggestions, and receive support from their fellow students in a safe environment. The Heidelberger anti-stress seminar learning objectives are summarized in Table 1 (Tab. 1) along with Bloom's taxonomy of learning objectives [23]. The detailed content of each seminar session is shown in Table 2 (Tab. 2).

#### Seminar evaluation 

Participants evaluated each seminar evening via questionnaire containing general questions comprising six recurring items (see Table 3 (Tab. 3), “General Evaluation”), which were posed after every seminar evening and referred to students’ general satisfaction with the seminar session, the setting, and the student tutors. In addition, the questionnaire comprised 3-6 further items specific to each seminar evening referring to learning content of the session. All questions were answered on a Likert scale ranging from 1 to 7 (1=strongly agree; 7=strongly disagree). In addition, students were able to evaluate the respective seminar with an overall school grade (1=very good to 6=unsatisfactory).

## Results

### Participant characteristics

From a total of 659 students (Year 1: n=321/year 2: n=338), 75 students participated (65%. female; mean age 20.5±3.3 years) in the peer-led stress seminars on a voluntary basis. In the first year of implementation, this corresponds to a number of 39 participants and 36 participants in the following year.

#### Student tutor characteristics

In 2013, a total of five female tutors from the third to fifth year of study held the seminars (mean age 25.0±2.3 years). In the following year (2014), three of the five students from 2013 held the seminars again, supplemented by two male tutors from the fourth and fifth year of study (mean age 26.8±2.6 years).

#### Evaluation results

The general and specific seminar evaluation data are shown in Table 3 (Tab. 3). The so far n=75 participants assessed the stress prevention program for first year medical students very positively. The seminar series was evaluated with a school grade of 1.2±0.4 in WS 2013/14 and with 1.5±0.5 in the following year. Particularly, the peer tutors’ competence was evaluated as very high (1.4 to 1.5 approval rate on the Likert scale). 

## Discussion

The Heidelberger stress prevention seminars were introduced in WS 2013/14 as an additional seminar on a voluntary basis for first year medical students and have since become a fixed part of the Heidelberg Curriculum Medicinale (HeiCuMed). The seminar evenings’ very good evaluations as well as the received personal feedback emphasize the seminars importance at the Heidelberger Faculty of Medicine. In our opinion, it is especially beneficial to offer medical students stress prevention programs during the demanding transitional phase at the start of their studies in which students must master the step into independent life. Especially during this phase of individuation - cutting the cord from the familiar environment while simultaneously dealing with overburden and lack of orientation in the new “system” - excessive self-demands and fear of failure can become prominent. The early on implementation of a primary preventive measure could offer the possibility to counteract the development of emotional exhaustion or cynicism at an early stage as already suggested by Yusoff [24], although proof of data is still lacking. Apart from the early on implementation of the preventive measure, the decision to opt for peer-led seminars following the PAL principle seems to have proven effective. The competence of the student tutors was particularly highlighted in the evaluations. Hence, corroborating our assumption that PAL can be effectively implemented in presumably more “sensitive” areas, such as for stress prevention, which may require particularly high interactivity and spontaneity on the part of the student tutor. Although the stress seminars primarily dealt with strategies for coping with the immediate academic requirements, it seems that participating students also benefited from the direct contact with the student tutors who themselves had only recently overcome the initial hurdles of medical studies. As a result, the question asking whether the students saw their tutors as possible role models received considerable affirmation (also see [25]). This observation corresponds to the constructs of the social and cognitive congruence between tutors and students [19], [20]. As, to our knowledge, this is the first description of PAL in the field of stress prevention at a medical school, the question of student tutor feasibility seems highly relevant to us. After all, a complex topic, such as stress management, could prove to be excessively demanding for the young teachers. Despite their “pioneer character”, our experiences show good feasibility after the first two years of implementing the stress prevention seminars. Of course, one should not disregard that the student tutors leading the seminars, though complete novices in the field of stress management, were all highly experienced in teaching in other fields in advance. It therefore seems essential to us, especially when initially unfamiliar with the topic, that the implemented student tutors have no additional “fear of contact” with the student teaching itself, which can only be ensured through focused and repeated training of the tutors pools [26]. Accordingly, the student tutors voiced a uniformly high level of satisfaction after having led the seminars. This is also reflected in the fact that three of the five tutors, which had held the seminars in 2013, agreed to lead them again in the following year. The evaluation of the individual seminar components shows that among the offered relaxation techniques mindfulness exercises (Mindfulness-Based Stress Reduction, MBSR) were experienced as most easily incorporated into students’ everyday life, whereas visualization methods, such as guided imagery exercises were experienced as difficult to integrate. Participants perceived the student tutors’ exercise instructions to be good or very good. The themed session dealing with coping strategies was experienced to be particularly helpful. Although stress management seminars are beneficial during medical studies, it should not go unmentioned that psychosocial issues, such as how to deal with study-specific stressors, should generally be given greater consideration in the context of university teaching in order to increase and substantiate the effect of this additive measure. In the long run, only breaking the taboo of and raising awareness for mental health problems will make the goal of improving medical students’ health achievable [27]. Of course, changes in content and structure of the medical degree program itself are necessary for the long-term reduction of (partially system-native) stress factors. Accordingly, medical school syllabus modifications, such as “streamlining” the curriculum through consecutive content reduction while increasing problem-oriented teaching, represent a significant cornerstone for structural prevention on the road to the sustained improvement of students’ quality of life [28], [29]. Hereby, the focus should lie on giving students more scope for the individual organization of their studies [30]. Especially, as the university environment and the reduction of study pressures have been proven to contribute to the reduction of health problems [31], [32], [29].

## Conclusions

The concept of PAL seems to be suitable for the field of stress prevention. Accordingly, student participants evaluated the seminars and student tutors very positively. The long term feasibility of this concept certainly depends on whether the stress seminars are able to achieve a sustainable effect. This should be the focus of specific efficacy studies in future.

## Ethics

The study was conducted following the Code of Ethics of the World Medical Association (Declaration of Helsinki 6th revision, 2008). Written informed consent was obtained from all participants as approved by the local Ethics Committee of the University of Heidelberg (No. S-396/2013).

## Funding

The study was funded by the Ministry of Science and Art of Baden-Württemberg as part of the “Center of Excellence for the Prevention of Psychological and Psychosomatic Disorders in the Working and Educational World” [Kompetenzzentrum zur Prävention psychischer und psychosomatischer Störungen in der Arbeits- und Ausbildungswelt].

## Acknowledgement

We would like to thank Anna Cranz for excellent proofreading.

## Competing interests

The authors declare that they have no competing interests.

## Figures and Tables

**Table 1:**
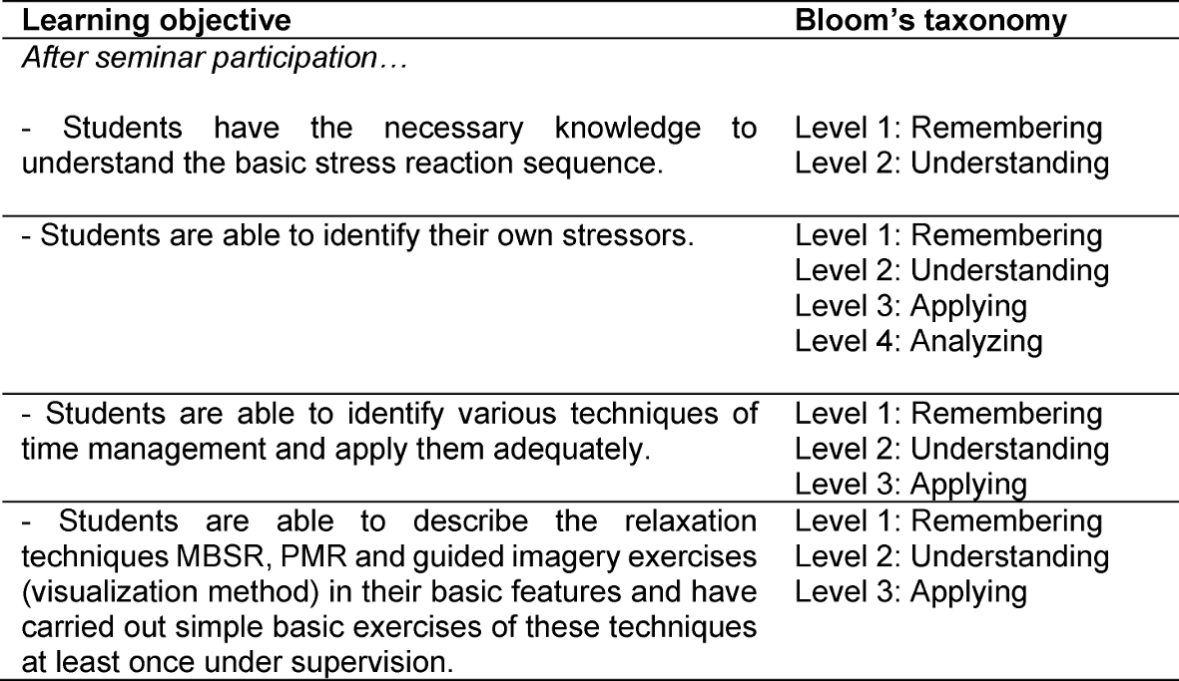
Learning objectives and the corresponding taxonomy by Bloom [23].

**Table 2:**
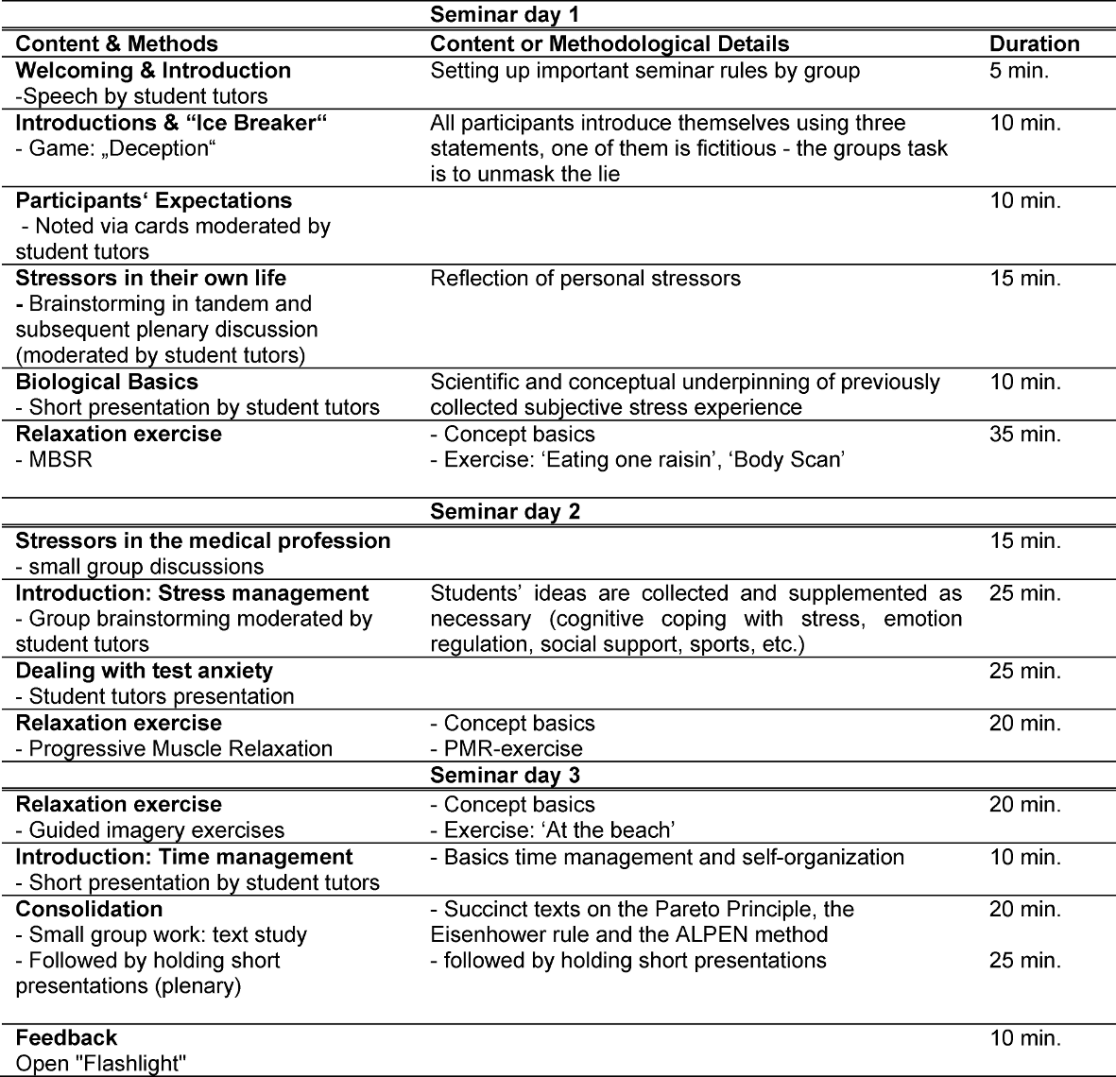
Seminar contents, methods, and setting information.

**Table 3:**
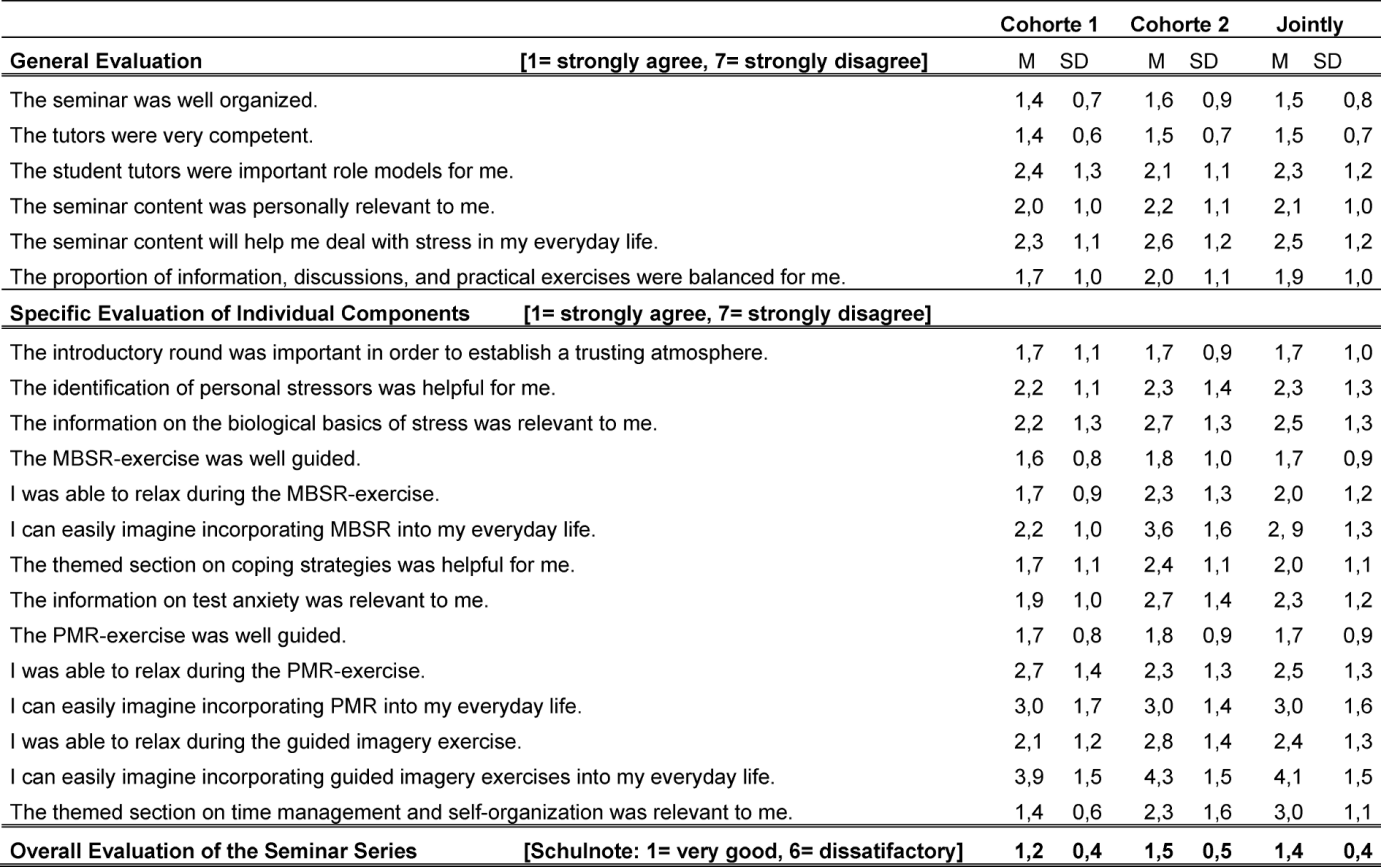
Evaluation results of the three seminar evenings for both cohorts (Cohort 1: WS 2013/14; Cohort 2: WS 2014/15).

## References

[1] Prins JT, van der Heijden FM, Hoekstra-Weebers JE, Bakker AB, van de Wiel HB, Jacobs B, Gazendam-Donofrio SM (2009). Burnout, engagement and resident physicians' self-reported errors. Psychol Health Med.

[2] Dyrbye LN, West CP, Satele D, Boone S, Tan L, Sloan J, Shanafelt TD (2014). Burnout among U.S. medical students, residents, and early career physicians relative to the general U.S. population. Acad Med.

[3] Voltmer E, Rosta J, Aasland OG, Spahn C (2010). Study-related health and behavior patterns of medical students: A longitudinal study. Med Teach.

[4] Scholz M, Neumann C, Steinmann C, Hammer CM, Schroder A, Essel N, Paulsen F, Burger PH (2015). Development and correlation of work-related behavior and experience patterns, burnout and quality of life in medical students from their freshmanship to the first state examination. Psychother Psychosom Med Psychol.

[5] Koehl-Hackert N, Schultz JH, Nikendei C, Möltner A, Gedrose B, van den Bussche H, Jünger J (2012). Burdened into the job -- final-year students' empathy and burnout. Z Evid Fortbild Qual Gesundheitswes.

[6] Maslach C, Jackson SE (1986). Maslach burnout inventory.

[7] Maslach C (2013). Die Wahrheit über Burn-out. Stress am Arbeitsplatz und was Sie dagegen tun können.

[8] Aster-Schenck IU, Schuler M, Fischer MR, Neuderth S (2010). Psychosocial resources and burnout risk factors in medical school: a cross-sectional study and analyses of need for preventive curricular interventions.. GMS Z Med Ausbild.

[9] Goebert D, Thompson D, Takeshita J, Beach C, Bryson P, Ephgrave K, Kent A, Kunkel M, Schechter J, Tate J (2009). Depressive symptoms in medical students and residents: a multischool study. Acad Med.

[10] Daly MG, Willcock SM (2002). Examining stress and responses to stress in medical students and new medical graduates. Med J Austr.

[11] Simard AA, Henry M (2009). Impact of a short yoga intervention on medical students' health: a pilot study. Med Teach.

[12] Erogul M, Singer G, McIntyre T, Stefanov DG (2014). Abridged mindfulness intervention to support wellness in first-year medical students. Teach Learn Med.

[13] Wild K, Scholz M, Ropohl A, Brauer L, Paulsen F, Burger PH (2014). Strategies against burnout and anxiety in medical education--implementation and evaluation of a new course on relaxation techniques (Relacs) for medical students. PloS one.

[14] Soriano RP, Blatt B, Coplit L, CichoskiKelly E, Kosowicz L, Newman L, Pasquale SJ, Pretorius R, Rosen JM, Saks NS, Greenberg L (2010). Teaching medical students how to teach: a national survey of students-as-teachers programs in U.S. medical schools. Acad Med.

[15] Yu TC, Wilson NC, Singh PP, Lemanu DP, Hawken SJ, Hill AG (2011). Medical students-as-teachers: a systematic review of peer-assisted teaching during medical school. Adv Med Educ Pract.

[16] Secomb J (2008). A systematic review of peer teaching and learning in clinical education. J Clin Nurs.

[17] Dandavino M, Snell L, Wiseman J (2007). Why medical students should learn how to teach. Med Teach.

[18] Ten Cate O, Durning S (2007). Dimensions and psychology of peer teaching in medical education. Med Teach.

[19] Lockspeiser TM, O'Sullivan P, Teherani A, Muller J (2008). Understanding the experience of being taught by peers: the value of social and cognitive congruence. Adv Health Sci Educ Theory Pract.

[20] Schmidt HG, Moust JH (1995). What makes a tutor effective? A structural-equations modeling approach to learning in problem-based curricula. Acad Med.

[21] Ledig T, Eicher C, Engeser P (2014). AaLplus – History Taking and Physical Examination – a Course for Preclinical Medical Students. Z Allg Med.

[22] Kadmon M, Strittmatter-Haubold V, Greifeneder R, Ehlail F, Lammerding-Koppel M (2008). The sandwich principle--introduction to learner-centred teaching/learning methods in medicine. Z Evid Fortbild Qual Gesundheitswes.

[23] Bloom BS, Englehart MB, Furst EJ, Hill WH, Krathwohl DR (1956). Taxonomy of Educational Objectives, the classification of educational goals – Handbook I: Cognitive Domain.

[24] Yusoff MS (2014). Interventions on medical students' psychological health: A meta-analysis. J Taibah University Med Sci.

[25] Nikendei C, Andreesen S, Hoffmann K, Obertacke U, Schrauth M, Junger J (2008). Final-year medical students as tutors for undergraduate students during their on-ward courses in internal medicine: a quantitative analysis. Z Evid Fortbild Qual Gesundheitswes.

[26] Heni M, Lammerding-Koppel M, Celebi N, Shiozawa T, Riessen R, Nikendei C, Weyrich P (2012). Focused didactic training for skills lab student tutors - which techniques are considered helpful?. GMS Z Med Ausbild.

[27] illis JM, Perry WR, Carroll EY, Hibble BA, Davies MJ, Yousef J (2010). Painting the picture: Australasian medical student views on wellbeing teaching and support services. Med J Aust.

[28] Slavin SJ, Schindler DL, Chibnall JT (2014). Medical student mental health 3.0: improving student wellness through curricular changes. Acad Med.

[29] Kohls N, Bussing A, Sauer S, Riess J, Ulrich C, Vetter A, Jurkat HB (2012). Psychological distress in medical students - a comparison of the Universities of Munich and Witten/Herdecke. Z Psychosom Med Psychother.

[30] Dyrbye LN, Thomas MR, Shanafelt TD (2006). Systematic review of depression, anxiety, and other indicators of psychological distress among U.S. and Canadian medical students. Acad Med.

[31] Dyrbye LN, Thomas MR, Harper W, Massie FS, Jr, Power DV, Eacker A, Szydlo DW, Novotny PJ, Sloan JA, Shanafelt TD (2009). The learning environment and medical student burnout: a multicentre study. Med Educ.

[32] Dyrbye LN, Power DV, Massie FS, Eacker A, Harper W, Thomas MR, Szydlo DW, Sloan JA, Shanafelt TD (2010). Factors associated with resilience to and recovery from burnout: a prospective, multi-institutional study of US medical students. Med Educ.

